# Methodological review: Prioritizing a future research agenda for overcoming immunization implementation barriers in Pakistan

**DOI:** 10.1016/j.puhip.2026.100723

**Published:** 2026-01-14

**Authors:** Wardah Ahmed, Asma Ummad, Majid Tahir, Alyssa Sharkey, Ahsanullah K. Bhurgri, Omera Naseer, Shifa Habib, Zahid Memon

**Affiliations:** a Health Policy and Management Section, Department of Community Health Sciences, Aga Khan University, Karachi, Pakistan; b National Institute of Health(NIH), Islamabad, Pakistan; c School of Public and International Affairs, Affiliate-Center for Health and Wellbeing, Associate- Office of Population Research, Princeton University, New Jersy, United States; d Technical Consultant, Health Department, Government of Sindh, Pakistan

**Keywords:** Priority setting, Methodological review, Immunization research, Pakistan

## Abstract

**Background:**

Research prioritization plays a critical role in aligning health research agendas with the urgent system-level challenges, especially in resource-constrained settings. This study aims to analyse the methodology of priority setting workshop, conducted to prioritize a future research agenda for overcoming immunization implementation barriers in Pakistan.

**Study design:**

A methodological review, a subtype of observational study designs.

**Methods:**

In June 2024, an immunization priority setting workshop was convened by the National Institute of Health (NIH) and Aga Khan University (AKU), and WHO-Alliance for Health Policy and Systems Research. Our review analyses the design, and implementation of the workshop methodology which integrated Dot Voting and Impact Feasibility Matrix and post-workshop Delphi-informed thematic categorization process. The prioritized research questions were via consensus among 26 subject matter experts from government, academia, and frontline program implementers.

**Results:**

The Dot Voting method surfaced both technical (e.g., data systems, serosurveys) and contextual (e.g., workforce motivation, coverage gaps) research questions, fostering layered domains. Impact Feasibility Matrix confirmed these priorities while surfacing underemphasized areas (e.g., cost-effectiveness and operational feasibility), thus enhancing methodological triangulation. Subsequent Delphi-informed process synthesized 23 highest-ranked questions synthesized into four themes: (1) Strengthening immunization program through improved data system, (2) Innovative digital health approaches to strengthen immunization system and data quality, (3) Enhancing workforce capacity and well-being in immunization program, (4) Ensuring fair access and distribution of vaccination services.

**Conclusion:**

This methodological review highlights the value of structured and participatory processes in identifying priority research areas for immunization implementation. However, their success depends not on just the process itself but also on being reflective, inclusive and able to manage complex challenges. Future, efforts in Pakistan and similar settings should balance real-world limitation with strategies to ensure equity and stakeholder engagement. This case adds to the global conversation on strengthening methods for setting health research priorities in low-middle-income countries.

**What this study adds:**

• Demonstrates the practicality of a hybrid, participatory research prioritization method to identify context-specific immunization research needs in Pakistan.
• Simple tools like voting and rating ideas based on impact and practicality can help make better decisions that match both local and global immunization priorities.
• Provides an example of how various stakeholders worked together to plan research goals in a clear and fair way that everyone agreed on in a limited time.

**Implications for Policy and Practice:**

• Institutionalize inclusive, participatory research-prioritization processes by incorporating policy makers, researchers, implementers, and tools to address power dynamics.
• Support integrated immunization research planning by assessing multidimensional immunization system needs.
• Strengthen transparency and consensus-building through iterative mechanisms to manage disagreement and revisit divergent views

## 1 Introduction

Research prioritization plays a critical role in aligning health research agendas with the urgent system-level challenges, especially in resource-constrained settings [1]. When well-executed priority setting methodologies ensure that future research efforts are directed towards projects that generate timely, actionable, and context-relevant evidence [2]. However, while the importance of inclusive, stakeholder-driven prioritization is widely acknowledged in global health, the methodological approaches to structuring and operationalizing stakeholder engagement in priority-setting exercises remain unexplored particularly in low-middle-income countries (LIMICs) [3,4]. The health system in fragmentation and competing policy demands present unique challenges in LMICs. A systematic analysis of such participatory methodologies can provide valuable insights into how collaborative approaches ensure that research efforts are well-targeted and impactful, ultimately contributing to the success of programs [1,5,6].

Most existing literature on health research prioritization has focused on what topics should be prioritized, rather than how those priorities should be identified through participatory methods. Frameworks such as the Child Health and Nutrition Research Initiative (CHNRI), and James Lind Alliance (JLA) protocols offer structured models for eliciting stakeholder input [1,7,8]. Yet, few studies critically examine their adaptability, feasibility, and reflexive application in LMIC programmatic contexts or under complex governance arrangements [3,6,9]. Moreover, while stakeholder engagement is increasingly emphasized in implementation research, its methodological rigor, inclusivity, and real-world application in national immunization programs remains insufficiently documented [3,5].

This methodological review addresses this gap by assessing a research priority-setting exercise conducted in Pakistan in 2024, situated within the global effort to institutionalized embedded implementation research (EIR). Pakistan presents a complex immunization ecosystem, characterized by regional and local disparities in health infrastructure, vaccine hesitancy, sociopolitical instability, and programmatic fragmentation [10,11]. Despite substantial global investment, immunization coverage remains uneven, in 2023, only 3 out of 14 vaccines had a coverage rate of 90 %with others, lagging as low as 40 % [12]. These challenges highlight the need for context-specific, methodologically grounded strategies to guide future research that can address persistent implementation barriers [13,14].

To meet this need, a national workshop was convened in June 2024 by the National Institute of Health (NIH), Aga Khan University (AKU), and the Alliance for Health Policy and Systems Research (HPSR)-WHO, under the umbrella of MAINSTREAM Initiative-a multicounty effort to institutionalize the EIR in GAVI-supported programs. This event brought together stakeholders from government, academia, frontline health program implementers to prioritize a structured research agenda targeting immunization delivery challenges in Pakistan [15,16]. This review provides a methodological analysis of that priority setting process, focusing on design, participatory tools, and embedded institutional collaboration. Specifically, the workshop employed a Dot Voting technique [17], Impact-Feasibility Matrix [18] and Delphi technique [19], adapted for the Pakistani immunization context. The hybrid approach sought to balance speed with depth, flexibility with structure and successful participatory goal-setting models in other LMICs. This triangulated approach aligns with successful applications of hybrid methodologies in similar LMIC health systems [9,20].

The workshop employed a systematic approach with three core purposes: (i) to identify key knowledge and implementation gaps in national and provincial immunization programs; (ii) to collaboratively define prioritization criteria grounded in both evidence and practitioner experience; and (iii) to develop replicable methodological framework for future priority setting initiatives in LMIC immunization system. The exercise was guided by research question; *How effective was this hybrid prioritization methodology in generating actionable, consensus-driven research priorities in a complex immunization policy environment like Pakistan?* This methodological review contributes to emerging literature on contextualized and participatory methods for research prioritization in global health. It reflects on the adaptability of existing tools, the operational challenges of participatory design, and the implications for embedding learning into national systems. Through this, we aim to inform future methodological innovation in stakeholder engagement for priority setting not only in Pakistan but across similar health systems.

### 1.1 Objective

This study analyzed the methodology of priority setting workshop, conducted to prioritize a future research agenda for overcoming immunization implementation barriers in Pakistan.

## 2 Methods

### 2.1 Study design

A methodological review, a subtype of observational study designs.

### 2.2 Workshop implementation design

The national research priority setting workshop was held in Islamabad, Pakistan in June 2024. The methodology employed a multi-phase, participatory design, incorporating three distinct but complementary methods:

1. Dot Voting
2. Impact feasibility Matrix
3. Post-workshop Delphi-informed thematic categorization

The hybrid methodology combining Dot Voting, Impact-Feasibility Matrix, and the Delphi technique was purposefully selected to address the complexity of immunization priority setting in Pakistan's diverse and evolving health system context [[17], [18], [19]]. There was also previous practice experience of NIH and AKU to conduct these workshops and also supported by literature. Each method contributed to synergistic strength; Dot Voting was employed to rapidly surface stakeholder preferences across a broad range of research topics, enabling inclusive and efficient initial filtering; the Impact-Feasibility Matrix was introduced to structure deliberation around both strategic value and practical implementation of ideas, facilitating evidence-informed yet grounded decision-making; Delphi technique provided a structured, iterative process for refining consensus among diverse participants including policymakers, program managers, and researchers across national and provincial levels.

### 2.3 Pre-workshop preparation

The preparatory phase of the workshop took place from May 20, 2024, to June 04, 2024. During this phase, a structured document review was conducted to collect and consolidate 52 potential research questions. This review included 25 questions from WHO immunization research agenda, 15 questions from GAVI's thematic focus areas, and 12 questions from the Joint External Evaluation (JEE) 2023 report of Pakistan. These questions were compiled and distributed to participants as a reference document at the start of the workshop. This pre-workshop synthesis served to both grounded discussions in existing knowledge and minimize duplication of effort during the participatory process. The project approval was obtained from the Ethical Review Committee of Aga Khan University.

### 2.4 Participant selection and inclusion

A total of workshop participants included 26 subject matter experts (SMEs). Selection criteria included nomination by respective departments and organizations, and active involvement in immunization programs and relevant public health initiatives in Pakistan. Our analysis considers the representativeness of the final participant composition, which included individuals delegated from the Federal Directorate of Immunization (FDI), Provincial and Regional Expanded Programs of Immunization (EPIs), the Drug Regulatory Authority of Pakistan (DRAP), the Polio Eradication Initiative (PEI), WHO, UNICEF, and academic institutions, such as the Federal Urdu University of Science and Technology (FUUAST) and the Health Services Academy (HSA). While this ensured broad institutional coverage, it also resulted in the underrepresentation of community-level implementers and frontline health workers. We consider how this stakeholder composition influenced the workshop dynamics and outcomes.

### 2.5 Workshop structure and facilitation

The workshop opened introductory presentations, followed by breakout group discussion. Participants were divided into three multidisciplinary groups, with each group tasked with reviewing a portion of the 52 research questions. Facilitators from NIH and AKU were trained in inclusive moderation techniques to ensure that all voices were heard and to minimize any personality dominance. While all participants had equal voting right, no formal observation or recording protocol was used to assess participation dynamics.

### 2.6 Dot Voting

In the first phase, participants used Dot Voting to prioritize research questions. Each participant received 13 dot stickers (25 % of total options i.e.52 questions) to distribute across any number of questions based on perceived importance. In total, 338 votes were recorded across all research questions. Flip charts were used for visibility, and each group presented their results to the plenary. This method offered a time-efficient and transparent mechanism to organize priorities. Its flexibility allowed participants to express their preferences by concentrating votes on high-value questions or distributing them across diverse issues.

### 2.7 Impact Feasibility Matrix

Following Dot Voting, participants assessed the top-ranked questions using an Impact Feasibility Matrix, facilitated through structured group discussion and a Google Forms tool. Questions were categorized into four quadrants based on:

• Impact: Likely effect on improving immunization outcomes
• Feasibility: Practicality of implementation, considering resources, geography, time, ethics and political context.

The resulting four quadrant classification system to categorize research questions: Quadrant A (high impact, high feasibility) which included priority research questions for implementation; Quadrant B (high impact, low feasibility) which included research questions considered valuable but requiring extensive resources and time due to their complexity; Quadrant C (low impact, high feasibility) which included research questions that would be easy to implement but offer minimal benefits and may be considered for elimination; and Quadrant D (low impact, low feasibility) which included research questions that should be eliminated.

### 2.8 Post-workshop Delphi-informed thematic categorization

After the workshop (next day), facilitators from AKU, NIH and focal person from Alliance WHO engaged in a Delphi-informed thematic categorization process. This step transformed a long list of prioritized items into a structure research agenda, aligning with the MAINSTREAM initiative's operational research objectives.

## 3 Results

### 3.1 Dot Voting results

**Fig. 1:**
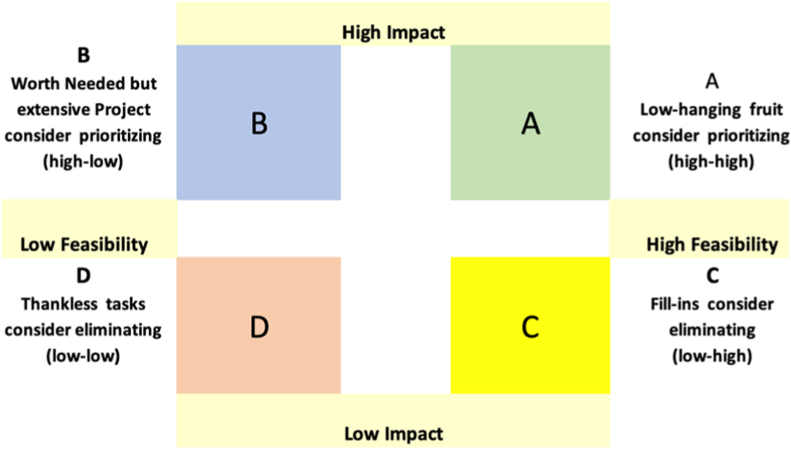
Dot voting.

The Dot Voting method (Fig. 1) enabled the surfacing of research questions that were both technical (e.g. data system, serosurveys) and contextual (e.g. workforce motivation, coverage gaps).

#### 3.1.1 Methodological assessment of the Dot Voting method

The Dot Voting methodology generated an initial ranked list of research priorities, demonstrating its utility as a participatory prioritization technique. Table 1 presents the top five research priorities identified through this method. Our reflection is that this methodology surfaced multifaceted research priorities that span technical challenges (data collection methods, technological limitations) and social/logistical barriers, and enabled identification of a long list of potential priority research topics.

**Table 1:** Top five research priorities identified by the Dot Voting Method.

Rank	Research Domain
1.	Identifying and addressing barriers to accurate denominator and numerator estimation
2.	Research on coverage surveys: recall, traceback, sampling, missing data, and wealth proxies
3.	Evaluate interventions for strengthening data-related workforce capacities and training
4.	Integrate immunization and Vaccine-Preventable Diseases (VPD) serosurveys
5.	Compare quality of life between vaccinated and non-vaccinated individuals for VPD

In addition, the Dot Voting approach facilitated the emergence of complex research domains rather than overly simplified questions. For example, the second priority research topic identified (“Research on coverage surveys”) encompassed multiple interconnected aspects of coverage surveys: recall validity, facility traceback, sampling methods, missing data handling, and wealth assessment proxies. This suggests the methodology captured nuanced research needs that would not have emerged through more restrictive prioritization approaches. The methodology also proved helpful in identifying a range of priorities (e.g., workforce issues versus innovative technical serosurveys).

### 3.2 Impact Feasibility Matrix results

**Fig. 2:**
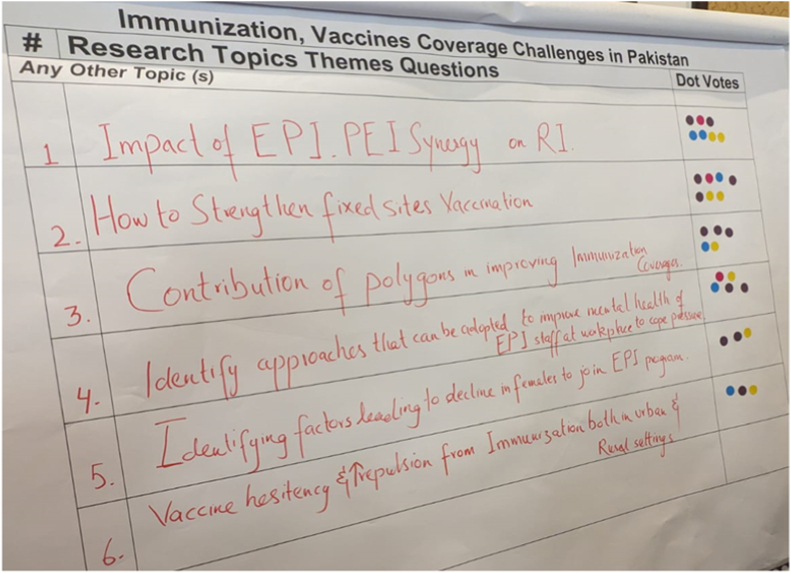
Impact feasibility matrix.

The matrix (Fig. 2) confirmed most of the Dot Voting priorities, suggesting methodological triangulation. However, it also surfaced previously neglected domains, particularly economic feasibility and cost-effectiveness, demonstrating its added value. This methodology provided a useful analytical framework for further refining research priorities beyond simple ranking and complementing the perspectives of all stakeholders.

#### 3.2.1 Methodological assessment of the Impact Feasibility Matrix

The Impact Feasibility Matrix methodology provided a useful analytical framework for further refining research priorities beyond simple ranking. Table 2 presents the top five priorities identified through this approach. The methodology's strength was evident in how it maintained consistency with four of the five priorities identified through the Dot Voting method, suggesting strong triangulation and validation of priorities across these two participatory approaches.

**Table 2:** Top five research priorities identified by the Impact Feasibility Matrix.

Rank	Research Domain
1.	Research on coverage surveys: recall, traceback, sampling, missing data, and wealth proxies.
2.	Evaluate interventions for strengthening data-related workforce capacities
3.	Integrate immunization and VPD serosurveys
4.	Compare quality of life between vaccinated and non-vaccinated individuals for VPD.
5.	Cost-effectiveness studies of feasible approaches in resource-constrained settings.

A notable finding was the emergence of a new priority (i.e., cost-effectiveness studies) that had not appeared in the Dot Voting results. This demonstrates how the two-dimensional assessment of impact and feasibility allowed for consideration of additional factors (in this case, economic considerations) that might have been overlooked in the Dot Voting approach. This suggests that using multiple prioritization techniques can add value by capturing different aspects of priority setting that might be missed by a single approach.

### 3.3 Post-workshop Delphi-informed thematic categorization

The final thematic structure helped communicate a clear research agenda. Its strength lies in synthesizing diverse priorities into actionable coherent groupings that maintained logical and underlying connections across the related research priorities aligned with national immunization strategy.

**Fig. 3:**
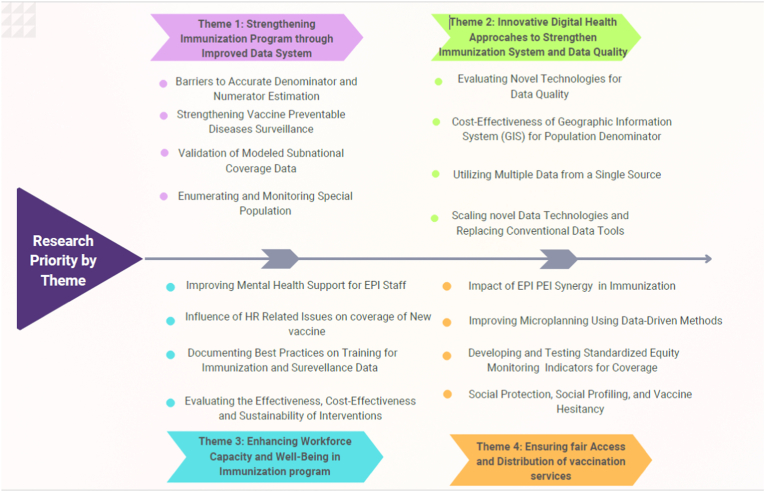
Prioritized research questions into thematic areas.

Over three iterative rounds, they grouped the 23 highest ranked research questions into four core themes (Fig. 3):

1. Theme 1: Strengthening immunization program through improved data system
2. Theme 2: Innovative digital health approaches to strengthen immunization system and data quality
3. Theme 3: Enhancing workforce capacity and well-being in immunization program
4. Theme 4: Ensuring fair access and distribution of vaccination services

## 4 Discussion

This methodological review examined the hybrid design and implementation of a priority setting workshop aimed at identifying research priorities for overcoming immunization implementation barriers in Pakistan. Structured, participatory and transparent processes can support policymakers and funding agencies in making informed investment decisions for global health research priorities [21,22]. Our analysis reveals important insights into both the strengths and limitations of the methodologies employed, with particular emphasis on their contextual applicability and potential for future adaptation.

The workshop methodology shared certain characteristics with established frameworks such as the Child Health and Nutrition Research Initiative (CHNRI) and the James Lind Alliance (JLA) approaches. The CHNRI framework typically employs a quantitative scoring of predefined criteria by technical experts, providing a high degree of analytical rigor and replicability. However, its reliance on scoring tools can be less adaptable to dynamic or emergent group discussions. In contrast, our approach emphasizes real-time deliberation and consensus-building which supported more context-responsive decision making [7,23].

The JLA model, meanwhile, emphasizes co-production of research agenda with patients and community members, often using nominal group techniques. While our process included a range of technical experts and policy makers, it lacked systematic engagement with community stakeholders [24]. This reflects a key limitation incorporating structured mechanisms from JLA such as preparatory community consultations or joint ranking exercises, could have improved the legitimacy and equity of the final priorities [8,24,25].

Therefore, the three methods Dot Voting, Impact-Feasibility Matrix, and the Delphi process, each contributed distinct insights, yet also revealed areas of alignment. Dot Voting generated a broad snapshot of participant preferences by identifying high-interest topics, often reflecting immediate programmatic concerns such as vaccine hesitancy, data quality, and workforce challenges. In contrast, the Impact-Feasibility Matrix introduced a more strategic layer by highlighting research questions that were not only important but also actionable within existing system constraints often shifting focus toward service integration and performance monitoring. Finally, the Delphi-informed thematic analysis brought a degree of thematic coherence by clustering overlapping questions and consolidating consensus across stakeholder groups. Notably, while there was alignment on certain themes (e.g., improving routine immunization coverage and addressing inequities), divergence appeared in the prioritization of more operational versus systemic issues. This divergence may reflect differences in how participants interpreted feasibility some favoring pragmatic interventions, others focusing on structural change. Literature on hybrid prioritization methodologies supports such triangulation as a strength, suggesting that diverse methods help surface latent tensions and deepen validity through iterative consensus [1,26]. Hence, the interplay between these methods added analytical depth, reinforcing rather than conflicting with each other's outputs.

While the workshop primarily engaged policy-level and technical stakeholders, it is crucial to acknowledge that community-perceived barriers to immunization may differ in emphasis or framing from system-level constraints. In the Pakistani context, communities often cite trust in health workers, religious beliefs, gender norms, and access to vaccination sites as critical barriers factors that may not receive sufficient weight in system-led prioritization [13,15]. For example, while policymakers might prioritize logistical challenges such as data management or surveillance integration, communities may perceive barriers more through the lens of service acceptability or interpersonal experiences with frontline providers. The lack of community representatives in the current priority-setting exercise may thus limit the perceived legitimacy or comprehensiveness of the priorities identified. Future iterations should incorporate participatory mechanisms such as community consultations or embedded qualitative surveys to better align priorities with lived experiences, particularly in regions with deep structural inequalities [27].

### 4.1 Strengths and limitations

The structure of workshop includes Dot Voting, Impact Feasibility Matrix and Delphi-informed thematic categorization enabled a coherent, efficient, and context relevant approach to prioritization enabled balanced consideration of both stakeholder preferences and implementation realities to drive meaningful change at the provincial level aligning with national and global immunization priorities. Additionally, the participation from diverse institutional representatives co-produced well organized research agenda (Supplemental file 1). The transparent use of voting and matrix tool added procedural clarity and encouraged participants to articulate their reasoning.

Despite these strengths, we identified few methodological limitation. While equal voting rights were intended participation, this did not fully mitigate power asymmetries in group discussions, particularly in a system marked by hierarchical norms in the local context [23]. Although the technical experts participated in voting exercise, aimed to identify the most urgent and need based research priorities thereby addressing the tension between achieving consensus and ensuring validity, the process lacked observation checklist or facilitation metrics [28]. Therefore, we cannot claim that all voices carried equal weight in the discussions. Furthermore, several questions could logically fit under multiple themes. While overlaps were acknowledged, no cross-thematic mapping or multi-tagging was performed.

Another limitation was not inviting of frontline health workers and affected communities in the priority setting workshop as stated above. This was treated as a logistical constraint rather than a methodological gap; this would be addressed by integrating validation follow up round via embedded survey or community consultation in the later program cycle.

In Pakistan, strong collaboration and leadership are essential for national level priority-setting processes in immunization to be transformative. These are also crucial for managing revisions, transparency, and fostering a unified approach to improving immunization coverage in the country [23,24,29,30]. In this experience and in spite of identified limitations, the workshop provides an opportunity to build rapport and establish a common understanding among a diverse set of stakeholders, enhancing group cohesion and productivity in terms of highlighting priority research questions based on immunization gaps.

### 4.2 Conclusion

This methodological review highlights the value of structure and participatory processes in identifying priority research areas for immunization implementation. However, their success depends not on just the process itself but also on being reflective, inclusive and able to manage complex challenges. Future, efforts in Pakistan and similar settings should balance real-world limitation with strategies to ensure equity and stakeholder engagement. This case adds to the global conversation on strengthening methods for setting health research priorities in low-middle-income countries.

## Data statement

The data (research material) is added as Supplemental file-1 Prioritization Themes and Topics.

## Ethical statement

The ethical approval was obtained from Ethical Review Committee (ERC) Aga Khan University (AKU)-2024-9822-28969. This work is part of the project “Institutionalizing learning by mainstreaming embedded implementation research in country immunization programmes (MAINSTREAM) in Pakistan.” The Aga Khan University selected as the MENTOR institute of this project in Pakistan.

## Declaration of generative AI and AI-assisted technologies in the manuscript preparation process

During the preparation of this work Wardah Ahmed used claude.ai in order to [correct the overall grammatical errors]. After using this tool/service, the author reviewed and edited the content as needed and took full responsibility for the content of the published article.

## Funding

This publication received financial support from the 10.13039/100007855Alliance for Health Policy and Systems Research. The Alliance is able to conduct its work thanks to the commitment and support from a variety of funders. These include our long-term support from the 10.13039/100004441Swedish International Development Cooperation Agency (Sida) and the 10.13039/100007843Norwegian Agency for Development Cooperation (Norad), as well as designated funding for specific projects within our current priorities. This publication was supported by Gavi, The Vaccine Alliance. For the full list of Alliance donors, please visit https://ahpsr.who.int/about-us/funders.

WHO Reference 2024/1464444-0.

## Declaration of competing interest

The authors declare no competing interests.

## Abbreviations

CHNRI: Child Health and Nutrition Research Initiative
EPI: Expanded program of immunization
JLA: James Lind Alliance
JEE: Joint External Evaluation
PEI: Polio eradication initiative
SME: Subject matter expert
